# Removal of a suture needle: a case report

**DOI:** 10.1186/s40902-021-00309-3

**Published:** 2021-07-05

**Authors:** Suyun Seon, Baek-Soo Lee, Byung-Joon Choi, Joo-Young Ohe, Jung-Woo Lee, Junho Jung, Bo-Yeon Hwang, Min-Ah Kim, Yong-Dae Kwon

**Affiliations:** Department of Oral and Maxillofacial surgery, School of Dentistry, Kyung Hee University, 26, Kyungheedae-ro, Dongdaemun-gu, Seoul, 130-701 South Korea

**Keywords:** Needle fracture, Suture needle, Iatrogenic foreign body, Foreign body removal, Oral cavity

## Abstract

**Background:**

Foreign bodies may be embedded or left behind in the oral cavity during oral surgical procedure. The loss of instruments such as impression material, surgical gauze, and broken injection needles are commonly reported in the dental field. These complications are generally symptomatic and show signs of inflammation, pain, and purulent discharge. Accidental breakage of suture needles is a rare but potentially dangerous event.

**Case presentation:**

In this report, we present one case of lost suture needle during the procedure of flap operation at local dental clinic and its successful removal under local/general anesthesia administration via CBCT with a help of two reference needles to localize the 6-0 nylon needle and consulting with the clinician.

**Conclusion:**

CT scanning taken while mouth-closing may not be accurate with regard to real location measurement performed while mouth-opening. If so, other up-to-date radiographic devices and methods to retrieve a needle are recommended.

## Background

Various suture materials are used in the intraoral surgery and frequently penetrate the soft tissue of the oral cavity. This surgery is usually associated with implant surgery, dental surgical extraction, periodontal flap surgery, oral-maxillofacial surgery including bone grafts, osteomyelitis, cancer operation, and operative trauma. Due to many factors such as limited intraoperative visibility and difficult access, sudden movement of patients, the size of the instruments, and mishandling of surgeons with lack of experiences, the materials and instruments used in oral surgery can be broken, displaced, embedded, and left behind during these surgeries. Patients with this condition often experience unpleasant symptoms such as pain in operation site, post-operative exudate, and swelling, and some of these foreign bodies that are adjacent to the vital anatomical structures can lead to serious complications such as massive bleeding, nerve injuries, and purulent inflammation leading to airway obstruction and suffocation. There are various ways recommended for the treatment of retained foreign body removal in the literatures. The key to removing foreign bodies in head and neck surgery is exact localization and surgical approaches. We report one successful case of removing the suture needle that is used in a flap surgery for bone graft with an assistance of CT image and two injection needles inserted as reference points to exactly localize and detect.

## Case presentation

**Fig. 1:**
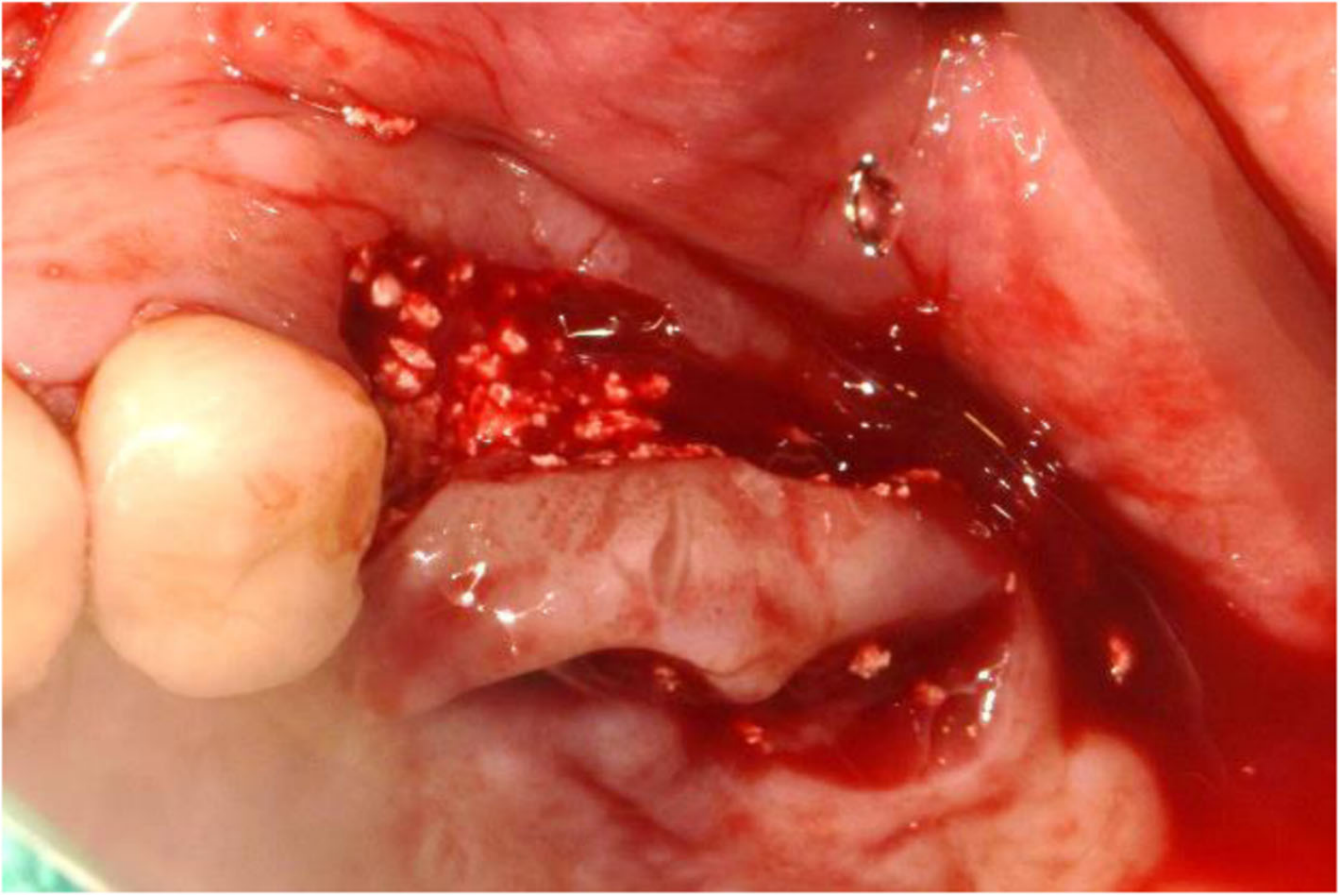
Clinical photo taken right after flap operation of explantation at the local dental clinic

A male patient with a past medical history of hypertension was referred by his dental clinician to our department to remove a suture needle in the left buccal mucosa. The patient had undergone explantation surgery with bone grafts on the #26i, 27i due to the chronic peri-implantitis in the local clinic. The procedure was accompanied by local flap surgery to cover the grafted bone material filling the extracted area for better bone formation and healing. The sutures were supposed to be tied with 6-0 nylon, but the suture needle was cut off during the procedure. Although the local clinic immediately attempted to retrieve the suture needle using additional panorama and CT, their attempt failed (Fig. 1).

**Fig. 2:**
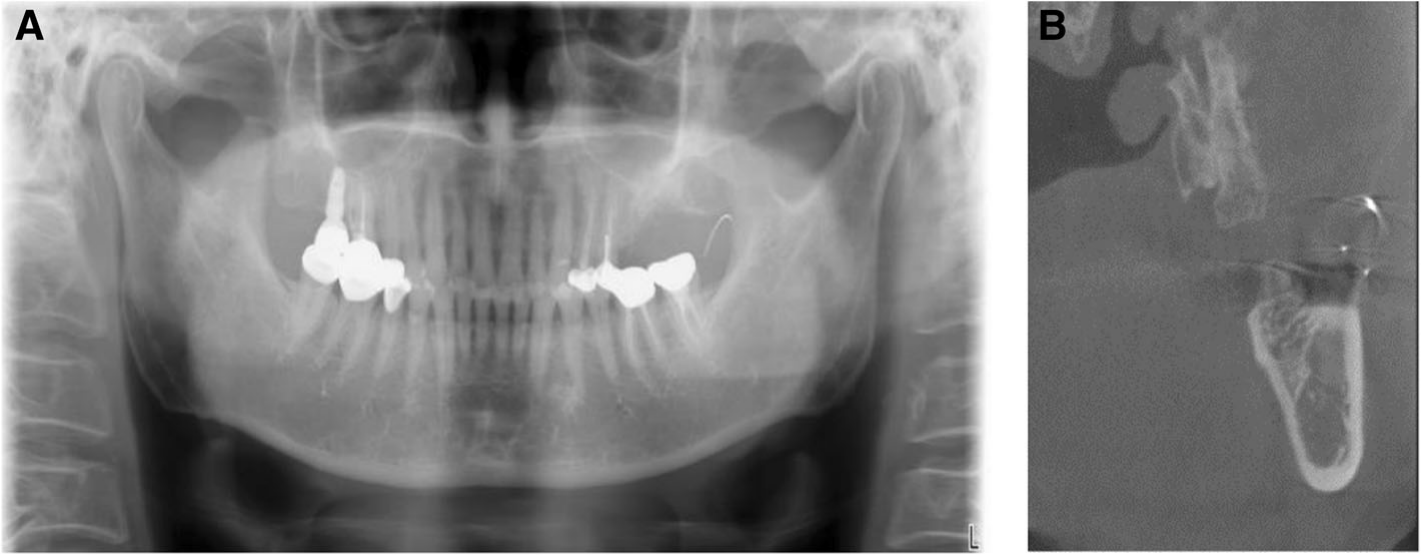
**A** Panoramic radiograph taken on arrival shows needle fragment located at the anterior ramus of left mandible. **B** CBCT image of the lost needle with 2 injection needles as reference points

Upon his arrival to our department without bringing any radiographs, the patient was complaining of sharp pain on the operation site with no limitation in his ability to open his mouth. Panoramic radiograph confirmed that the suture needle still stayed in the left buccal space, anterior to the ramus of mandible (Fig. 2A). The oral examination with palpation was performed, and two injection needles were inserted almost perpendicular to each other into the left buccal mucosa near the anterior border of mandibular ramus in order to locate the needle as reference points. Cone beam computed tomography was performed and revealed that the broken needle was located above the injection needle and below the maxillary tuberosity (Fig. 2B).

It was decided that surgical exploration under local anesthesia was the most appropriate way to remove the broken needle. A vertical incision on the left buccal mucosa along the ramus was made, and the blunt dissection using a dissecting scissor was performed. The needle, however, was invisible, and the first attempt failed due to the patient’s pain.

Following discussion with patient and patient’s caregiver about the possible risks of migrating the needle into deeper layers and damaging anatomical vital structures with the further exploration under local anesthesia, they agreed to the retrieval of the suture needle under general anesthesia.

**Fig. 3:**
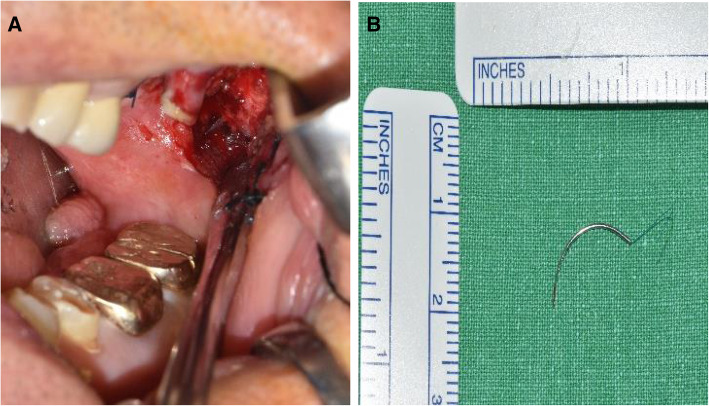
**A** A single needle fragment embedded in the buccal mucosa. **B** The suture needle successfully retrieved

On the following day, the patient underwent the surgical exploration under general anesthesia. Taking the clinician’s information that the initial suture was performed on the left upper second molar region into consideration, the additional vertical incision was made on the left buccal mucosa at the height level of upper occlusal plane. Careful exploration began by blunt dissection with curved and straight mosquitoes to expose the deep soft tissue layers. Further exploration was performed, and the broken 6-0 nylon round needle was identified in one piece (Fig. 3A). The needle was approximately 1.3 cm long, and the nylon fragment was attached to it (Fig. 3B). After the retrieval of the needle, the mucosal incision was closed up using 4-0 Vicryl. Post-operative radiographs were taken afterwards and confirmed that the broken needle was successfully removed. 1 week later, the stitches were removed, and the patient had progression of healing on the surgical site without any infection or complication.

## Discussion

The loss of surgical needles is estimated to be a quite common occurrence during a surgical procedure especially in the surgical departments of the medical field, while reports in the dental field for a loss of needles are relatively scarce. Jayadevan et al. [1] reported 65% of medical surgeons in their survey conducted in 2014 experienced lost surgical needles in the USA and designed a protocol to recover the lost needle during minimally invasive surgery.

Previous studies have reported accidental injection needle fractures and successful retrieval. However, suture needle events tend to be rare and less reported in the oral surgical field (Table 1). Blum et al. [32] reported 100 cases of needle breakage occurred from 1914 to 1928 and Catelani et al. [18] reported 82 cases of broken anesthetic needles since 1965. Augello et al. [33] reviewed the literature of the last 50 years and reported 70 percent of needle breakage events occurred with inferior alveolar nerve block anesthesia in the pterygomandibular space. Although the incidence of needle fracture has decreased due to the introduction of stainless and flexible metal alloys, many cases of needle fracture have been constantly reported without any consensus on methods to retrieve the needle fragments or the guidelines on how to remove the lost needle.

**Table 1:** Recent cases of needle removal

Authors (year)	Type of needle	cases	Site of needle fragment	Detection method	Required time	Successful removal
McDonogh (1996) [2]	Injection needle	1	Retromolar region (right)	DPR, oblique lateral, lateral skull. RG KEELER metal detector	1 h	Yes
Bhatia (1998) [3]	Injection needle	1	Pterygo-mandibular space (left)	PA RG	2 h	Yes
Zeltser (2002) [4]	Injection needle	1	Pterygo-mandibular space (left)	DPR, PA RG, CT, 23G needle soaked in methylene blue	Immediately	Yes
Thompson (2003) [5]	Injection needle	2	Pterygo-mandibular space (right, left)	RG, image intensifier with 2 reference needles	Immediately	Yes
Ethunandan (2007) [6]	Injection needle	1	Pterygo-mandibular space (right)	DPR, PA mandible, lateral cephalometric RG, CT	6 months	Yes
Dojcinovic (2007) [7]	Injection needle	1	Pterygo-mandibular space (left)	DPR, radioscopy	Immediately	Yes
Nezafati (2008) [8]	Injection needle	1	Pterygo-mandibular space (right)	CT, 18G needle, digital C-Arm	2 days	Yes
Pogrel (2009) [9]	Injection needle	16	Medial pterygoid muscle (left) (n=1), not defined (n=15)	DPR, CT, intra-operative RG with 2 spinal needles		Yes
Chrcanivic (2009) [10]	Injection needle	1	Pterygo-mandibular space (left)	Lateral skull RG	Immediately	Yes
Augello (2009) [11]	Injection needle	1	Masseter (right)	DPR, CT, fluoroscopy	Immediately	Yes
Hassani (2010) [12]	Suture needle	1	Tuberosity hamular-notch region	DPR, CBCT	4 years	Yes
Sencimen (2010) [13]	Suture needle	1	Pterygo-mandibular space (left)	DPR, CT, C-arm fluoroscope (Philips Medical Systems)	Few days	Yes
Prado (2010) [14]	Injection needle	1	Pterygo-mandibular space (right), migration to skull base	CT		No
Rifkind (2011) [15]	Injection needle	1	Pterygo-mandibular space (right)	DPR, CT, intra-operative fluoroscopy with 2 reference needles	8 days	Yes
Bacci (2012) [16]	Injection needle	1	Anterior part of temporalis muscle	DPR, fluoroscopy	Immediately	Yes
Brucoli (2012) [17]	Injection needle	1	Pterygo-mandibular space (left)	DPR, fluoroscopy	1 month	Yes
Catelani (2013) [18]	Injection needle	4	Pterygo-mandibular space (left)	CT, fluoroscopy (C-arm with 2 reference needles, methylene technique)	Immediately	Yes
					Immediately	Yes
			Extra-corporeal		Immediately	
			Pterygo-mandibular space (right)	CT, fluoroscopy (C-arm with 2 reference needles, methylene technique)		Yes
Gerbino (2013) [19]	Injection needle	1	Pterygo-mandibular space (left)	DPR, CT, navigation system (BrainLAB)	1 day	Yes
Kim (2013) [20]	Injection needle	1	Between coronoid process and condyle neck area(left)	DPR, CT		Yes
Nicot (2013) [21]	Injection needle	1	Subangulo-mandibular region	DPR, CT	5 days	Yes
Rahman (2013) [22]	Injection needle	1	In the vicinity of the lingula (right), migration to postauricular area	DPR, lateral oblique RG, CT	2 weeks	Yes
Ribeiro (2014) [23]	Injection needle	1	Pterygo-mandibular space (left), migration to medial wall of external auditory canal	CT	> 12 month	Yes
Acham (2014) [24]	Injection needle	1	Mandibular notch	DPR, CBCT, C-arm with 2 reference needles	6 h	Yes
Bailey (2015) [25]	Injection needle	1	Pterygo-mandibular space (right)	DPR, PA mandibular RG	Immediately	Yes
Casey (2015) [26]	Injection needle	1	Adjacent to the internal carotid artery	CT	4 years	Yes
Lee (2015) [27]	Injection needle	1	Pterygo-mandibular space above lingula (right)	DPR, CT, navigation system (Medronci AxiEM)	1 year	Yes
Okumura (2015) [28]	Injection needle	1	Parapharyngeal space adjacent to external carotid artery (right)	CT, facial x-ray, fluoroscopy, K-wire as reference bar	1 month	Yes
Stein (2015) [29]	Injection needle	1	Pterygo-mandibular space (right)	CBCT, navigation system (Medtronic StealthStation S7)	Immediately	Yes
Aktop (2015) [30]	Suture needle	1	Nearly below the medial pterygoid plate	DPR, CBCT	Immediately	Yes
Queiroz (2016) [31]	Injection needle	1	Cervical region close to facial artery	CT, C-arm image intensifier	Few days	Yes

DPR, CT, CBCT, PA, and RG represent dental panoramic radiography, computed tomography, cone beam computed tomography, posterior-anterior, and radiography respectively

Archer et al. [34] and Kennett et al. [35] reported management of broken needles using radiographs such as lateral and posterior-anterior views to locate the broken needles. As the panoramic radiograph has improved, preoperative diagnosis has become accurate and the surgical success has increased. Since the early 2000s, many authors reported the use of computed tomography for removal of the needles, and three-dimensional imaging techniques (CBCT) have become standard for localization and treatment strategies with the advantage of visualizing not only foreign bodies but also surrounding anatomical structures [4, 20, 24, 29]. Other techniques like fluoroscopy and mobile digital C-arm were introduced and suggested as reliable radiographic tools for needle retrieval [8, 13]. Keeping up with the latest technology, recent radiographic development illuminates the surgical navigation system to be the sophisticated tracking equipment as described by Lee and Zaid [27], Gerbino et al. [19], and Stein [29].

Many reports have described and/or recommended the use of reference needles placed in situ with additional radiographic images taken repeatedly for an effective localization of the needle [5, 8, 9, 11, 15, 16, 18, 24, 36–38]. Thompson et al. [5] reported that the use of two venipuncture needles can be successfully applied as reference needles.

Various methods to retrieve the fragment in the maxillofacial region were attempted and described in the past literatures. Although magnet received noticeable attention when introduced by Cohen [39] in 1963, other several authors reported that the use of magnets to determine the position and remove the fragment was not an ideal option since the hypodermic needles have a weak response to magnets [6]. Then, a metal detector was tried by McDonogh [2] and Okumura et al. [28] showing successful retrieval of an embedded broken needle.

Although CBCT is considered a precise method of imaging the location of a broken object in relation to adjacent anatomical landmarks, it is difficult to correlate the actual position with CT images owing to different intraoperative head and mouth-opening position compared to the mouth-closing position of CT images. Park et al. [40] described that CT scanning may not be accurate with regard to real position due to intraoperative traction and swelling and reported that the dental mini C-arm device was more beneficial in determining and confining the location of broken objects with intraoperative real time information. On this report, possible migration of the suture needle during the surgery made the situation challenging and the measurement on the CBCT scans was not helpful. Therefore, other up-to-date radiographic devices can be sometimes advisable when CT scan images are not practical. It should also be noted that surgeons sufficiently consult with the clinician who refers the patients by exchanging the information of the patient for the better diagnosis and accurately locating the lost objects in the oral cavity.

## Conclusion

This study reported a rare clinical case of removing the suture needle with the help of CBCT images. CT scanning taken while mouth-closing may not be accurate with regard to real location measurement performed while mouth-opening. If so, other up-to-date radiographic devices and methods to retrieve a needle are recommended.

## Data Availability

Non applicable

## Abbreviations

CBCT: Cone beam computed tomography
CT: Computed tomography
